# Structuring Continual Learning Through a Hierarchy of Objectives: A Conceptual Framework

**DOI:** 10.1177/23779608251389301

**Published:** 2025-11-25

**Authors:** Alette H. Svellingen, Kari Røykenes, Guttorm Brattebø

**Affiliations:** 1 Faculty of Health Sciences, 87368 VID Specialized University, Bergen, Norway; 2 Department of Clinical Medicine, 72984 University of Bergen, Haukeland University Hospital, Bergen, Norway

**Keywords:** healthcare, continual learning, conceptual model, a hierarchy of objectives, multiple learning activities, simulation-based education

## Abstract

**Background:**

Continual learning is fundamental for developing critical thinking and problem-solving skills. Although learning activities are well established in education, the connection between objectives, activities, and learning outcomes is often underemphasized. This article proposes a conceptual model that clarifies how learning processes can be better aligned through a hierarchy of objectives, using nursing education as an illustrative context.

**Methods:**

A conceptual analysis was conducted, drawing on established theories, including Kolb's Experiential Learning Theory, the Sociocultural Learning Perspective, and the NLN Jeffries Simulation Theory, to synthesize five core components: hierarchy of objectives, participant, learning activity, learning cycle, and multiple learning activities.

**Results:**

The model presents a structured approach to continual learning by clarifying how each component interacts to foster competence development. The model is presented from a general perspective while also specifically providing examples for the healthcare context. Simulation is used to exemplify how learning activities can support long-term learning goals.

**Conclusions:**

Understanding the importance of each level of the model and how these levels interact, can assist organizations in pursuing excellence. Accordingly, knowing and defining learning outputs and outcomes is the basis for attain the overarching goals. Facilitating learners to conceptualize reflections into active experimentation requires multiple learning activities, towards a spiral of continual learning.

## Introduction

**Figure 1.**
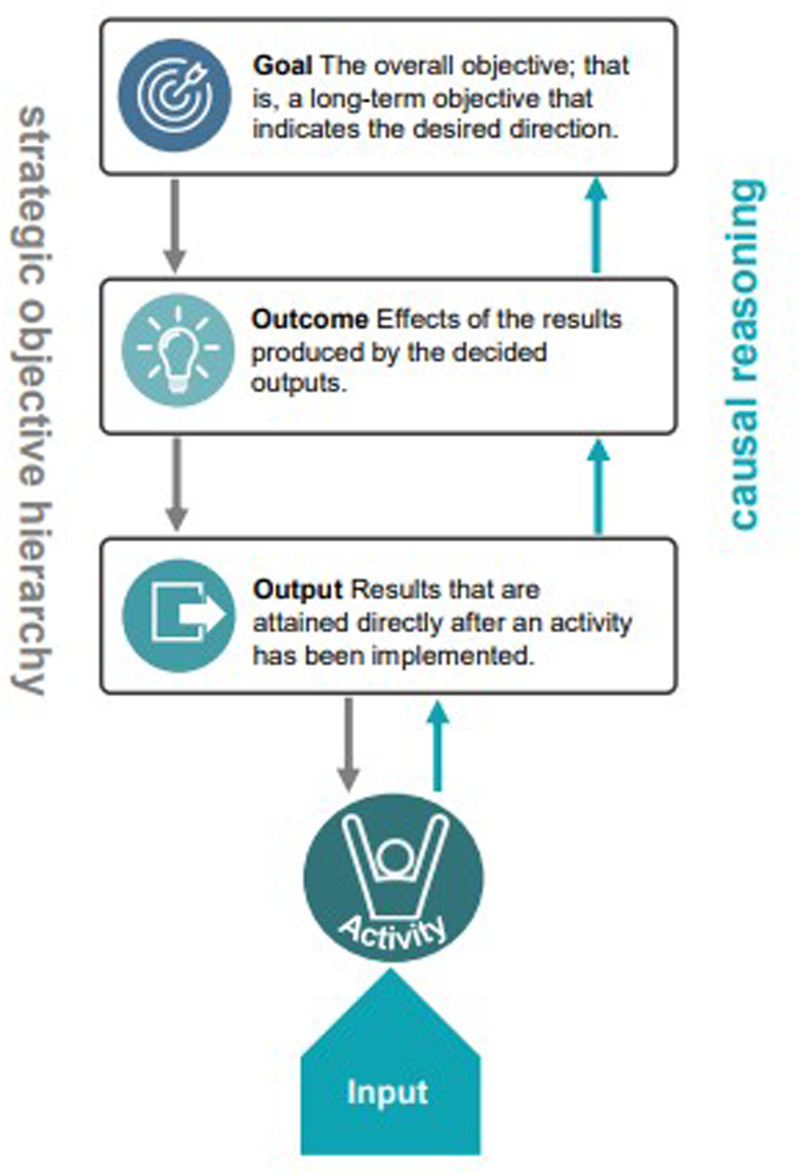
A Hierarchy of Objectives for Achieving Outputs, Outcomes, and Goals.

Keeping knowledge up to date, maintaining relevant skills, and staying motivated are essential for individuals in any professional field (Allen et al., 2024; Mlambo et al., 2021). A common approach to structuring and guiding development efforts is the use of a hierarchy of objectives (March et al., 1993; Ogbeiwi, 2021). Figure 1 illustrates how objectives at different levels are connected, providing the foundation for the model developed in this article. Outputs typically reflect immediate results, while outcomes capture broader, short-term performance (March et al., 1993; Ogbeiwi, 2021).

In nursing education, the primary objective is to ensure that students attain competence by engaging in meaningful learning activities (Song & McCreary, 2020). Although much attention has been paid to the design of learning activities, the role of hierarchical goalsetting in continual learning processes remain underexplored (Ogbeiwi, 2021). This gap can hamper students’ ability to connect learning activities to broader professional competency goals.

Educational programs often plan learning activities without fully considering their alignment with intended outputs and outcomes. Further, there is a tendency to focus on short-term achievements while overlooking their alignment to long-term developmental aims. A hierarchy of objectives can serve as a guiding structure for aligning immediate learning tasks with long-term outcomes. This perspective is particularly relevant in nursing education, where curricular design often spans both foundational training and continual learning (Ogbeiwi, 2021; Song & McCreary, 2020).

In the present study, continual learning is defined as the iterative progression of knowledge and skill acquisition within the timeframe of professional education, with nursing education serving as a paradigmatic example. Although the model may inform lifelong learning paradigms, its primary focus is to articulate mechanisms that support competence development during learning activities.

Drawing on existing theories and literature, this article aims to develop a conceptual framework that connects continual learning processes in nursing education to a hierarchy of educational objectives, to better understand how learning activities can be aligned with professional competence outcomes.

## Background

A fundamental way to enhance nursing students’ knowledge acquisition is to engage them in learning activities that bridge the gap between theory and practice (Greenway et al., 2019). Theoretical approaches such as Kolb's Experiential Learning Theory (Kolb, 2015), the Sociocultural Learning Theory (Lave & Wenger, 1991), and the NLN Jeffries Simulation Model (Jeffries & Rodgers, 2021) offer distinct yet complementary perspectives on learning processes. Kolb emphasizes transformation of experience into knowledge, Lave and Wenger highlight learning through social interaction, and Jeffries foregrounds simulation as a structured, learner-centered activity (Jeffries & Rodgers, 2021; Kolb, 2015; Lave & Wenger, 1991).

Simulation-based education (SBE) grounded in these theories has proven effective in bridging classroom learning and clinical practice, improving both immediate outputs and longer-term outcomes (Diaz & Anderson, 2021; Hanshaw & Dickerson, 2020; Saragih et al., 2024; Vangone et al., 2024). While each theory contributes valuable insights individually, their integration within a structured framework, such as a hierarchy of objectives, may offer a more systematic way to link learning activities with competence development across time. Existing models address various aspects of engagement, reflection, and teaching strategy (Froneman et al., 2022; Hattie & Donoghue, 2016; Tsimane & Downing, 2020). However, few explicitly incorporate a hierarchy of objectives or connect theoretical models to both short-term learning outputs and long-term outcomes. Connor et al. (2023) and Patel & Metersky (2022) explore clinical judgement and reflective practice yet stop short of positioning these elements within a broader strategic framework. Connor et al. (2023) conducted an evolutionary concept analysis of clinical judgment in nursing, aiming to develop a contemporary and operational definition of the concept that reflects current practice. Similarly, Patel and Metersky (2022) examined reflective practice in nursing, underscoring its significance in facilitating experiential learning and enhancing the quality of patient care. Tsimane and Downing (2020) proposed a model for facilitating transformative learning in nursing education, highlighting a learner-centered approach that prioritizes active engagement and personal development. In parallel, Froneman et al. (2022) presented a conceptual framework to support nurse educators teaching large classes, focusing on educator presence and reflective strategies to enhance student engagement. Hattie and Donoghue (2016) offered a conceptual model of learning that maps learning strategies to different stages of the learning cycle, helping educators support students’ progression. While these contributions provide valuable perspectives on pedagogy, motivation, and engagement, they stop short of systematically linking their proposed strategies to broader educational goals or a structured hierarchy of objectives.

The literature underscores the need for a more structured approach to competency development, one that integrates multiple learning theories to enhance the quality and relevance of nursing education (Lavoie et al., 2018). Although various models offer insights into specific aspects of teaching and learning, there remains a gap in conceptual frameworks that systematically connect learning activities to both immediate outputs and long-term professional outcomes. This creates a need for a model that clarifies how educational efforts contribute not only to short-term achievements but also to sustained competence essential for professional practice. This article addresses this gap by proposing a conceptual model that synthesizes key learning theories within a hierarchy of educational objectives, offering a structured perspective on how learning activities support the progressive development of professional competencies.

## Methods

This study employs a conceptual review methodology to synthesize theoretical knowledge and propose an integrative framework for competence development (Hulland, 2020; Jaakkola, 2020). This approach was selected to bridge established learning theories with the hierarchy of objectives framework.

### Search Strategy and Selection Criteria

A systematic literature search was conducted in PubMed, CINAHL, and Scopus using Boolean operators (AND, OR) to combine keywords such as learning, healthcare, nursing, nurse education, conceptual review, conceptual framework, and conceptual model. The search focused on English-language publications related to learning in nursing or health professions education. Titles and abstracts were screened for relevance. Inclusion criteria comprised theoretical and conceptual papers addressing learning theories, models, or frameworks. Empirical studies were included if they contributed conceptual insights. Studies unrelated to education or outside health professions were excluded.

### Thematic Analysis

Analysis proceeded in three stages: (1) identification of key learning theories and conceptual models; (2) collaborative extraction of recurring themes including continual learning, active learning, SBE, and learning activities; and (3) synthesis of these findings into a conceptual model. Themes were identified through iterative coding and discussion, guided by theoretical relevance and saturation. Analytical decisions were made collaboratively by all authors, drawing on their combined expertise in healthcare education and curriculum development.

**Figure 2.**
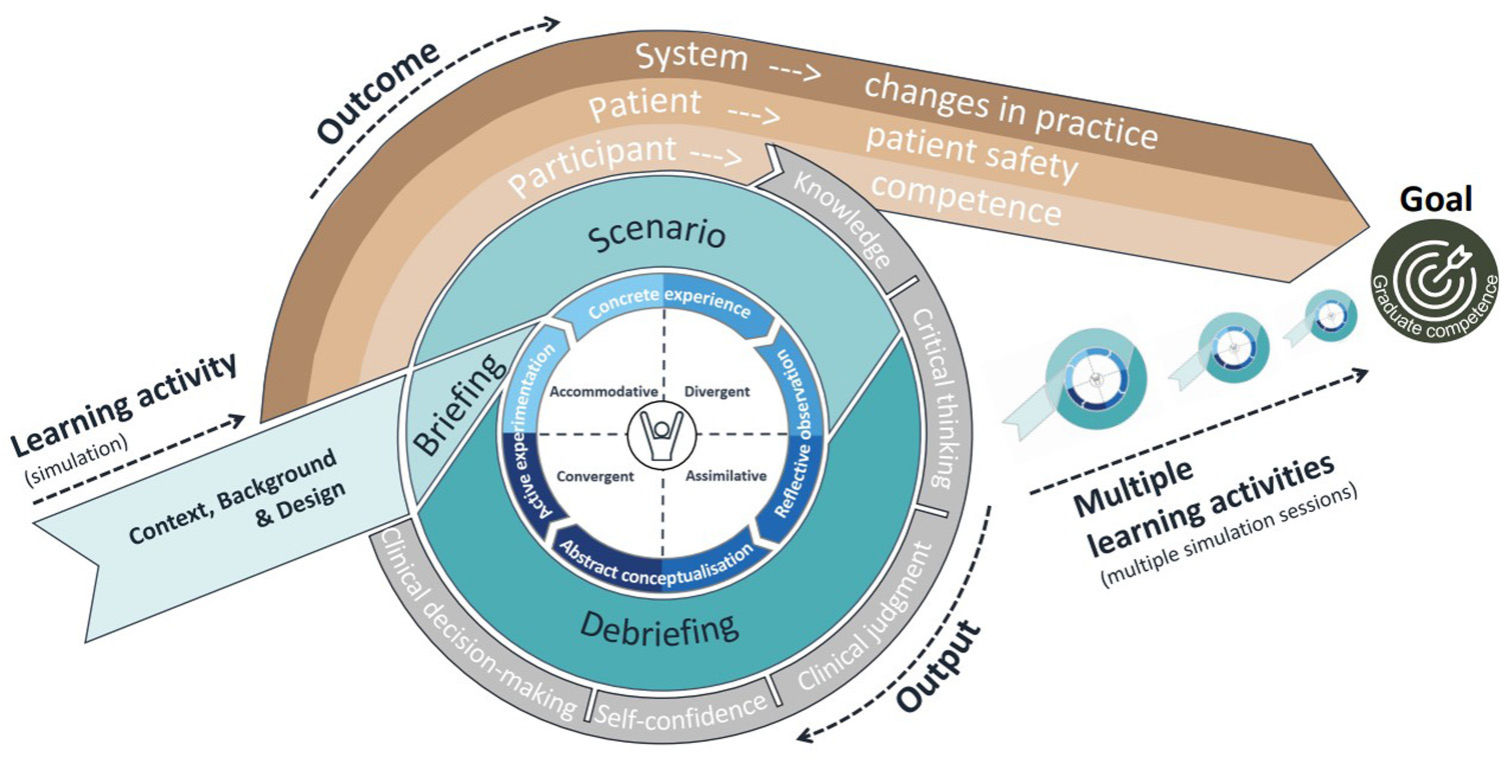
A Conceptual Model for Continual Learning Based on a Hierarchy of Objectives.

The analytic process involved comparing the contributions of each theory to learning and competence development, identifying overlaps and distinctions, and synthesizing them into five integrated components. These components were structured to reflect their function within a hierarchy of objectives and their connection to outcomes in SBE. While Figure 1 illustrates an established hierarchy of objectives used as a conceptual foundation, Figure 2 visualizes the novel conceptual model that emerged from the analytic synthesis. Appendix 1 provides a summary of the theoretical and empirical sources supporting each component of the model.

## Results

This article presents a conceptual model for designing and evaluating educational activities that foster professional competence in healthcare. The model integrates a hierarchy of objectives with three complementary learning theories to explain how structured learning activities foster learning outcomes. The NLN Jeffries Simulation Theory provides a framework for designing effective learning activities. Experiential Learning Theory explains how learners transform experience into knowledge, and the Sociocultural Learning Perspective emphasizes learning through participation in social interaction. Together, these elements are used to construct a coherent model that links educational intention to impact.

### Developing a Conceptual Model

A hierarchy of objectives provides the structural foundation for this model. It organizes goals at three levels, goal, outcome, and output, and highlights their causal interconnections (March et al., 1993; Ogbeiwi, 2021). Moving upward in the hierarchy addresses *why* an objective is pursued, while moving downward clarifies *how* it can be achieved (see Appendix 2). In educational contexts, this hierarchy frames learning outcomes as the result of systematically aligned processes (Selvik et al., 2022), supporting a system-thinking approach to curriculum design.

The NLN Jeffries Simulation Theory provides a guide for designing, implementing, and evaluating simulation sessions (Jeffries & Rodgers, 2021). High-quality simulation design is necessary to attain meaningful learning results (Beroz, 2021). Simulation sessions consist of three key elements: briefing, scenario, and debriefing, in which the facilitator plans and adapts the context, background, and design to optimize learner engagement and outcomes (Jeffries & Rodgers, 2021). As an experiential and learner-centered activity, simulation fosters competence and supports outcomes related to participants, patients, and healthcare systems (Jeffries & Rodgers, 2021). Multiple simulation sessions can progressively increase knowledge, critical thinking ability, clinical judgment, clinical decision-making, and self-confidence (Al Gharibi et al., 2020; Svellingen et al., 2020). Importantly, graduate competencies result from engaging in multiple complementary learning activities over time.

Learning theories illuminate essential aspects of simulation-based learning (Jeffries & Rodgers, 2021; Kolb, 2015; Lave & Wenger, 1991). The Experiential Learning Theory (Kolb, 2015), widely cited across professional education fields (Monteiro & Sibbald, 2020; Motta & Galina, 2023; O’Connor et al., 2022; Williams & Sembiante, 2022), conceptualizes learning as a dynamic process where individuals acquire knowledge by engaging in a cycle of concrete experience, reflection, conceptualization, and application.

Meanwhile, the Sociocultural Learning Perspective (Lave & Wenger, 1991; Vygotsky, 1978) emphasizes the importance of participation in communities of practice. Learning, according to this view, occurs through interaction within social and organizational contexts, gradually equipping individuals with the competencies and values needed to contribute meaningfully to their professional communities.

In this article, the hierarchy of objectives (March et al., 1993), the Experiential Learning Theory (Kolb, 2015), NLN Jeffries Simulation Theory (Jeffries & Rodgers, 2021), and the Sociocultural Learning Perspective (Lave & Wenger, 1991) are integrated into a conceptual model. Five central components emerged through the integration of theory and strategic logic: hierarchy of objectives, learning activity, learning cycle, participant, and multiple learning activities. Each was selected based on its theoretical grounding and functional role in fostering sustained professional learning, particularly in SBE. Together, these five components represent a minimal yet sufficient structure for modelling how competence is developed through practice-oriented learning. Each reflects a distinct but interconnected phase, from goal setting and instructional design to learner engagement, reflection, and cumulative development over time. These components and their interrelations form the conceptual model developed in this study, which is summarized in Figure 2.

The five components are grounded in a synthesis of both theoretical and empirical literature. As detailed in Appendix 1, each component is linked to core conceptual foundations and supported by research illustrating its educational function. The literature is grouped thematically, highlighting, causal reasoning, experiential learning, social participation, and learner engagement and collectively informs how the model integrates outputs, outcomes, and the long-term development of professional competence.

In the following, each layer of the model is described, starting from the outer edge and moving inward, first in general terms and then in relation to healthcare education. While the model is grounded in SBE, its structure can be adapted to other practice-oriented learning settings that aim to develop competence.

### The Conceptual Model

#### Hierarchy of Objectives

The hierarchy of objectives in this model links the activity to the overarching goal of graduate competence. The activity must be tailored to participants’ needs while aiming toward defined outputs and outcomes (Miller et al., 2021). In healthcare education, the goal is to develop graduate competencies, the integrated set of capabilities that students are expected to demonstrate at the end of their education. Outcomes refer to the development of participant competencies over time, such as clinical reasoning, communication, and decision-making. Outputs are the immediate, measurable learning results from specific activities, such as gains in knowledge, critical thinking, clinical judgment, decision-making, and self-confidence (Svellingen et al., 2020). This layered structure supports causal reasoning, guiding activity design toward long-term learning goals (March et al., 1993; Ogbeiwi, 2021). It provides the structural logic that connects individual learning actions to program-level goals through a chain of intended effects.

While the model centers on individual learning, outputs also carry conceptual relevance for patient safety and changes in practice at a system level. These broader effects are not directly assessed within the model but are important contextual considerations. Desired outputs and outcomes related to patient care and system improvement must be contextually defined and aligned with program aims to ensure that graduate competencies contribute meaningfully to healthcare quality.

#### Learning Activity

The learning activity is the central mechanism through which change occurs. It acts as the operative link between pedagogical design and the activation of the learning cycle, setting the development of competence in motion. The learning activity is the central mechanism through which change occurs. It acts as the operative link between pedagogical design and the activation of the learning cycle, setting the development of competence in motion. SBE exemplifies this alignment: the facilitator designs the learning environment, scenario, and debrief to achieve specific outputs (Diaz & Anderson, 2021; Jeffries & Rodgers, 2021). The activity activates the learning cycle, supports competence development, and fosters collaborative learning through participation, reflection, and dialogue (Hustad et al., 2019; Lave & Wenger, 1991; Watson et al., 2025; Wijnen-Meijer et al., 2022).

The learning activity functions as the engine of the model, driving the process of competence development. This role is not merely metaphorical, it is supported by empirical research demonstrating that well-designed learning activities, such as simulation, lead to measurable improvements in participants outcomes (Saragih et al., 2024; Stenseth et al., 2025).

#### The Learning Cycle

The learning cycle is essential to transforming activity into learning. Based on Experiential Learning Theory (Kolb, 2015), it consists of four interrelated stages: concrete experience, reflective observation, abstract conceptualization, and active experimentation. By guiding learners through reflection, conceptualization, and experimentation, the cycle transforms active participation into internalized competence.

The learning cycle offers a structured understanding of how learners transform experience into knowledge. The cycle has been shown to enhance not only individual cognitive outcomes, but also critical thinking, decision-making, and adaptability, skills that are essential for competence development in dynamic healthcare environments. To achieve the desired learning outputs, it is essential that all phases of the learning cycle are supported and completed within the activity. Each stage contributes uniquely to the transformation of experience into applied professional competence (Watson et al., 2025; Wijnen-Meijer et al., 2022).

SBE is particularly well suited to support this process, as it provides structured learning environments in which participants can progress through all four stages in a single session (Jeffries & Rodgers, 2021). In simulation, this cycle typically unfolds through briefing, scenario, and debriefing, giving participants opportunities to act, reflect, analyze, and reapply concepts in a safe yet realistic setting. When repeated across multiple learning activities, the cycle supports progressive integration of knowledge and promotes long-term competence development (Svellingen et al., 2020).

#### The Participant

At the center of the model is the participant, who enters the learning activity with motivation, prior knowledge, and a preferred learning style (Kolb, 2015). Kolb's four learning styles, accommodative, divergent, convergent, and assimilative, reflect different ways participants engage with the learning cycle: experiencing, reflecting, thinking, and acting. Entry requirements for healthcare education mean participants are generally highly motivated and engaged. However, participant backgrounds vary: some have relevant clinical experience, while others do not (Svellingen et al., 2021b). As a result, competency levels within a group can be diverse. The participant's prior knowledge and motivation may shape how effectively the learning cycle is completed and how learning outputs are achieved.

#### Multiple Learning Activities

Engaging in multiple learning activities provides participants with varied experiences that help them absorb and apply new knowledge. Through multiple exposure to learning activities, they can identify patterns and apply insights across different contexts (Kolb, 2015). Each activation of the learning cycle helps move participants from output toward outcome, bringing them closer to the goal. Over time, this multiple engagement transforms the learning cycle into a spiral of learning (Al Gharibi et al., 2020; Kolb, 2015; Svellingen et al., 2020). This cumulative process gradually moves learners from discrete outputs toward sustained outcomes and the overarching goal of graduate competence.

## Discussion

The conceptual model establishes connections among key components of learning. The model's components are interlinked: the participant shapes how the Learning Cycle is completed, the Learning Activity activates the cycle and connects it to the Hierarchy of Objectives, and Multiple Learning Activities build on prior cycles, advancing learning toward outcomes and goals. Each component is interdependent; removing any part would reduce the model's ability to help participants apply learning objectives across levels. Additionally, the model demonstrates how implementing multiple learning activities can accelerate continuous learning and foster the development of learning communities.

Learning is a dynamic process through which participants encounter, coordinate, and apply knowledge (Kolb, 2015). Exposure to multiple learning activities is essential to improve the learning curve, as it facilitates integration between prior experiences and new insights. A hierarchy of objectives, when paired with a participant-centered design, can lay the foundation for a functional learning community.

Traditionally, learning has been described through input–output models, but these often simplify the complexities of the educational process (March et al., 1993; Molloy et al., 2021). The proposed conceptual model addresses these limitations by introducing a cyclical perspective: learning is not simply a transfer from input to output, but a continuous, iterative cycle. Each component of this cycle must be activated for participants to connect new experiences with existing knowledge structures.

Causal reasoning refers to identifying possible cause-and-effect relationships, and the hierarchy of objectives applied in the conceptual model underlines the importance of each level and how they impact one another to achieve the learning goal (Miller et al., 2021; Ogbeiwi, 2021). In the top-down approach, the organization is guided by overarching objectives. Clearly defining outputs and outcomes is therefore essential for aligning activities with the organization's goals. Learning activities that do not correspond with these defined objectives are misaligned and less effective. Achieving meaningful learning outcomes requires that each learning activity is carefully planned and aligned with defined goals. This includes adapting the activity to the learners’ level, the specific context, and the overall design of the session. Attention to context, background, and design ensures that the learning experience is structured, relevant, and capable of fostering the intended competence development.

Students’ preferred learning styles may influence how they benefit from different components of the learning cycle, including learning through SBE (Svellingen et al., 2021a). Concrete learners, for example, benefit most when challenged to link concrete experiences to abstract conceptualization. For optimal outcomes, these learners must move beyond partial cycles focused on experience and engage with the full learning cycle (Kolb, 2015). Conversely, abstract learners can strengthen their decision-making and problem-solving skills by immersing themselves in the practical components of learning activities.

The learning spiral illustrates how previous knowledge and experience can be developed and transferred to new situations, promoting professional competence (Hung et al., 2021; Mancini et al., 2019). As learners move through repeated cycles, they refine their perspectives, transitioning between concrete and abstract thinking. This progression supports deeper understanding both individually and collectively (Raisch et al., 2018).

As participants become more aware of how they learn, their movement through the learning cycle can evolve into a spiral, accelerating their development (Diaz-Agea et al., 2021). This acceleration supports intrinsic motivation and curiosity, fostering continuous learning (Bächtold et al., 2023). The model may also increase nursing students’ motivation (Deci & Ryan, 2000) by assisting them conceptualize their reflections into active experimentation and think like professional nurses. Moreover, engaging in multiple learning activities can increase learners’ motivation by offering opportunities for personal growth (Diaz-Agea et al., 2021).

Continual learning is considered as a hallmark of professionalism (Laal et al., 2014). Professionals who pursue continuous development report greater job satisfaction and a stronger sense of fulfilment (Kind & Evans, 2015). Implementing relevant learning activities can help foster the ongoing learning necessary for individuals to become continual learners (Afonso et al., 2014; Hakvoort et al., 2022). Being part of a community of practice can support the internalization of continual learning as a professional value.

The transition from student to practitioner is often described as challenging (Hampton et al., 2021; Opoku et al., 2021). This shift requires learners to expand their approaches by engaging with the full cycle of experiential learning, especially the more practical dimensions. In this context, multiple simulation-based learning activities form a spiral of development that contributes to shaping a professional identity. This identity includes both competence and a sense of belonging (Adib-Hajbaghery & Sharifi, 2017; Connelly et al., 2023; Lave & Wenger, 1991). Novice healthcare providers need the courage to voice concerns when patient safety is at risk (Kruk et al., 2018); in the learning spiral, multiple simulation sessions increase such confidence (Svellingen et al., 2020; Turner & Harder, 2018). While the model was developed with SBE in mind, its relevance extends to leadership and team training in healthcare and other disciplines. By defining hierarchical objectives, designing targeted learning activities, and engaging participants through the learning cycle, educators can focus on both immediate outputs and long-term outcomes that support continual learning. Similar principles apply in technical and creative fields, where learning activities can activate the cycle of experience, reflection, conceptualization, and application. The model's core structure, linking learning activities to outputs, outcomes, and long-term goals, can be adapted to various professional settings, substituting simulation with other active learning strategies as needed. While some elements are already present in current educational practice, resource constraints can lead to uneven emphasis on key aspects. The model provides a structured framework that integrates these elements systematically, supporting a coherent and sustainable approach. This rationale extends beyond simulation and healthcare, helping safeguard continual learning and competence development in technical, creative, and organizational contexts.

The conceptual model builds on established theories, but it is essential to address their limitations. Kolb's Experiential Theory may oversimplify complex professional learning processes by focusing predominantly on individual reflection, without fully addressing social, cultural, and power dimensions. Similarly, Lave and Wenger emphasize social participation but offer limited insight into individual agency and the influence of structural constraints within communities of practice. These limitations point to the need for continued theoretical development and empirical validation. Future research should explore how the model functions across varying contexts and disciplines and consider longitudinal approaches to better capture how competence develops over time.

### Strengths and Limitations

A strength of this study is its conceptual approach, integrating selected, widely validated learning theories into a coherent framework. By aligning the hierarchy of objectives with learning activities, outputs, and outcomes, the model provides clarity for educators and curriculum developers and can be adapted across diverse educational contexts.

At the same time, the model is based on strategically selected learning theories rather than a systematic literature review. The aim was to support model development, not to map the full range of theory, and relevant perspectives may therefore have been omitted. The model also simplifies complex learning processes within a hierarchical structure, highlighting key relationships but not fully capturing their contextual, social, or emotional dimensions.

## Implications for Practice

Institutions should embrace the concept of continual learning, recognizing the potential of using a hierarchy of objectives to guide educational strategies. To effectively foster learning outcomes, educators should demonstrate a solid understanding of this hierarchy, enabling them to select and design learning activities that contribute to the learning process.

Although developed with initial nursing education in mind, the model may also be adapted for use in other educational and professional development settings by aligning goals, outcomes, outputs, and learning activities to the specific context. While the model is grounded in SBE, it is flexible: simulation activities can be replaced with other forms of practice-oriented learning, and each level of the objective hierarchy can be adapted to the specific educational context.

To strengthen learning and internalize key principles within a discipline, participants need to engage in activities that bridge theory and practice. Institutions should continuously revise and align their learning activities with overarching goals, cultivating a culture of curiosity, reflection, and innovation. As active members of a community of practice, educators and learners alike play a role in shaping institutional culture. By embracing continual learning, they can contribute to the development of learning organizations that support long-term professional growth.

## Conclusion

This conceptual analysis introduces a model that integrates key learning theories with empirical insights, offering a new perspective on continual learning. The structured approach promotes clarity in goal setting by aligning short-term outputs and outcomes with long-term objectives. A hierarchy of objectives comprising output, outcome, and goal, is essential for facilitating coherent and effective learning. Engagement in multiple learning activities supports participants in progressing from reflection and thinking to action and experiential learning. The model illustrates how continual learning, structured around a hierarchy of objectives, can enhance student and professional expertise and support ongoing professional development.

## Supplemental Material

sj-docx-1-son-10.1177_23779608251389301 - Supplemental material for Structuring Continual Learning Through a Hierarchy of Objectives: A Conceptual Framework Supplemental material, sj-docx-1-son-10.1177_23779608251389301 for Structuring Continual Learning Through a Hierarchy of Objectives: A Conceptual Framework by Alette H. Svellingen, Kari Røykenes and Guttorm Brattebø in SAGE Open Nursing

sj-docx-2-son-10.1177_23779608251389301 - Supplemental material for Structuring Continual Learning Through a Hierarchy of Objectives: A Conceptual Framework Supplemental material, sj-docx-2-son-10.1177_23779608251389301 for Structuring Continual Learning Through a Hierarchy of Objectives: A Conceptual Framework by Alette H. Svellingen, Kari Røykenes and Guttorm Brattebø in SAGE Open Nursing
