# Supplementary material for: Structuring Continual Learning Through a Hierarchy of Objectives: A Conceptual Framework
Source: SAGE Open Nurs. 2025 Nov 25;11:23779608251389301. doi: 10.1177/23779608251389301 (PMC12647548; doi:10.1177/23779608251389301)
Supplement: sj-docx-1-son-10.1177_23779608251389301 - Supplemental material for Structuring Continual Learning Through a Hierarchy of Objectives: A Conceptual Framework [file sj-docx-1-son-10.1177_23779608251389301.docx]

# Appendix 1: Overview of Theoretical and Empirical Sources Supporting the Conceptual Model

| **Component of the Model** | **Key Theoretical Foundations** | **Empirical References** | **Role in Model** |
| --- | --- | --- | --- |
| Hierarchy of Objectives | Ogbeiwi (2021); March et al. (1993) | Miller et al. (2021) | Links outputs, outcomes, and goals through causal reasoning |
| Learning Cycle | Kolb (2015); Dewey (1938) | Diaz & Anderson (2021);  Motta & Galina (2023); O’Connor et al. (2022); Williams & Sembiante (2022) | Frames experiential learning as a dynamic process |
| Simulation-Based Education (SBE) | Jeffries & Rodgers (2021) | Al Gharibi & Arulappan (2020); Diaz & Anderson (2021); Miller et al. (2021); Monteiro & Sibbald (2020); Svellingen et al. (2020) | Demonstrates practical implementation of the learning cycle |
| Multiple Learning Activities | Kolb (2015); Lave & Wenger (1991) | Al Gharibi & Arulappan (2020); Hustad et al. (2019) | Supports spiral learning and progression from outputs to outcomes |
| Participant Focus | Kolb (2015); Deci & Ryan (2000) | Al Gharibi & Arulappan (2020); Hustad et al. (2019); Svellingen, Røykenes et al. (2021) | Emphasises learner motivation, background, and engagement |
| Continual Learning & Professional Identity | Lave & Wenger (1991); Laal et al. (2014) | Adib-Hajbaghery & Sharifi (2017); Connelly et al. (2023) | Anchors the model in lifelong learning and identity formation |
| Sociocultural Perspectives | Vygotsky (1978); Lave & Wenger (1991); Engeström (2001) | Daniels (2001); Wertsch (2007) | Emphasizes social learning, cultural tools, and the importance of community and identity in professional development |
