# Supplementary figures and images for: Structuring Continual Learning Through a Hierarchy of Objectives: A Conceptual Framework
Source: SAGE Open Nurs. 2025 Nov 25;11:23779608251389301. doi: 10.1177/23779608251389301 (PMC12647548; doi:10.1177/23779608251389301)

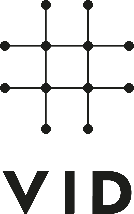

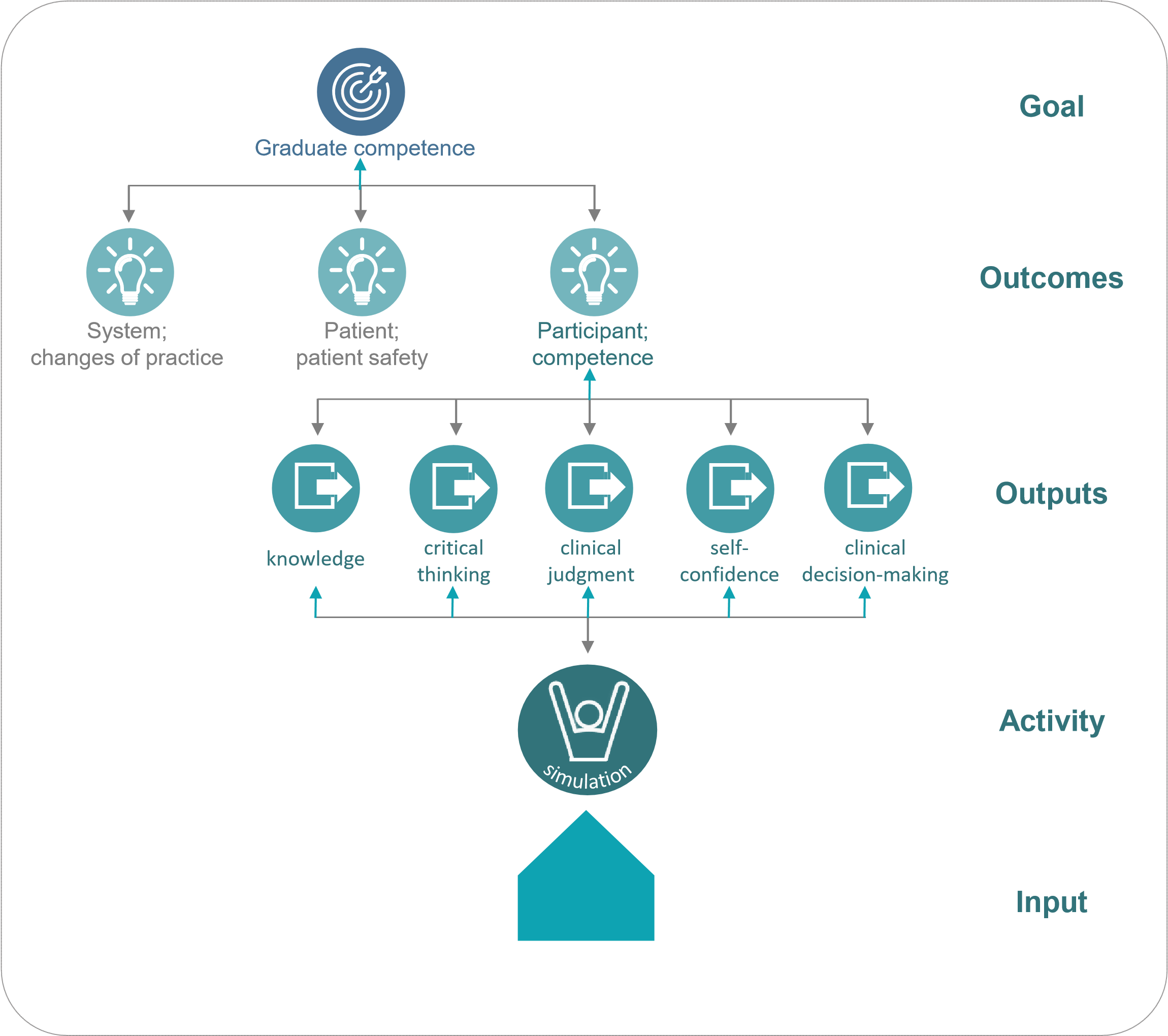

Supplement: sj-docx-2-son-10.1177_23779608251389301 - Supplemental material for Structuring Continual Learning Through a Hierarchy of Objectives: A Conceptual Framework [file sj-docx-2-son-10.1177_23779608251389301.docx]
